# Exploring Different Patterns of Love Attitudes among Chinese College Students

**DOI:** 10.1371/journal.pone.0166410

**Published:** 2016-11-16

**Authors:** Xianglong Zeng, Yiqin Pan, Han Zhou, Shi Yu, Xiangping Liu

**Affiliations:** Department of Psychology, Beijing Normal University, Beijing, China; University of Westminster, UNITED KINGDOM

## Abstract

Individual differences in love attitudes and the relationship between love attitudes and other variables in Asian culture lack in-depth exploration. This study conducted cluster analysis with data regarding love attitudes obtained from 389 college students in mainland China. The result of cluster analysis based on love-attitude scales distinguished four types of students: game players, rational lovers, emotional lovers, and absence lovers. These four groups of students showed significant differences in sexual attitudes and personality traits of deliberation and dutifulness but not self-discipline. The study’s implications for future studies on love attitudes in certain cultural groups were also discussed.

## Introduction

### Love Attitude and Its Measurement

Love has been typically defined as an emotional and passionate experience between two individuals [1], which may be reflected in several facets, such as attitude, emotion or behavior [2]. To analyze the complex concept of love and describe different types of romantic relationships, several love theories have been proposed [3]. One of the earliest and most influential theories is the colors of love, which was proposed by Lee in 1973. In this theory, there are three “primary colors” of love: eros or passionate love, which refers to the forceful physical or emotional attraction following commitment to a loved one; ludus or game-playing love, which represents the playful love felt by someone who has no commitment towards love or his/her partner; and storge or friendship-based love, which describes an intimate relationship developed gradually from prior friendship [4]. The three primary colors could combine in pairs and create three “secondary colors” that have their own particular properties and characteristics [5]: pragma or obsessive love, which is a combination between ludus and storge, refers to realistic and practical love that is not based on intense physical attraction but emphasizes the conscious search for a compatible partner; mania or possessive love, which is a combination between eros and ludus, is an obsessive, intense, full-feeling and possessive type of love held by lovers who have a strong need to be loved; and agape or altruistic love, which is a combination between eros and storge, refers to people who need attention from loved ones without having personal interest [6].

Compared with other love theories, the colors of love theory can provide a more comprehensive description of different attitudes in romantic relationships. For example, the triangular theory of love proposes that love consists of three components: intimacy, passion and commitment [7]. However, some types of love in the colors of love theory, such as ludus, in which a person considers that a relationship is a game without emotional attachment or commitment, are included in the theory. Because we want to explore the different attitudes held toward romantic relationships and are less concerned with debate over the definition of love, this article discusses romantic relationships from the point of view of Lee’s theory, which provides a more comprehensive description of romantic relationship.

Based on Lee’s theory, Hendrick and Hendrick [8] developed the Love Attitudes Scale (LAS). Empirical data confirms that the six dimensions in the LAS (storge, agape, mania, pragma, ludus and eros) are consistent with the six types of love in the colors of love theory. The difference between the two theories is that the former considers six dimensions as separate styles without distinguishing between primary and secondary. In addition, this scale evaluates respondents along all six dimensions, rather than categorizing them into sample profiles of six types of love. Based on the scores on each subscale, the propensity of an individual toward a certain love style can be determined [9].

### Individual Differences in Love Attitudes in Asian Culture

Love is considered a cultural construct [10]. Previous cross-cultural studies that compared Asian (i.e., Chinese, Japanese) and Western (i.e., American, British) people found that, generally, people in Asian cultures had a greater tendency to engage in pragma, whereas Western people showed a greater tendency toward eros or passionate love [9]. For example, Hendrick and Hendrick [8] found that Asian students in America had lower scores on eros and higher scores on storge and pragma compared with Black or White Americans. Dion and Dion [11] also found that Asians students scored higher on storge than Anglo-students in Canada. Goodwin and Findlay [12] also found that Chinese students were less engaged in eros but more so in agapic and pragma than British students. In addition to student samples, the study of Sprecher and Toro-Morn [13] on a general adult sample also confirmed that Chinese people were more likely to show storge and mania than Americans. Wan, Luk and Lai [14] further indicated that such patterns of cultural difference between Chinese and Western people may reflect the influence of the Confucian ethical system and collectivist beliefs.

One of the potential limitations of the cross-cultural studies mentioned above is that they mainly concerned the characteristics of love attitudes of Asian people in comparison with those of Western people and lacked an exploration of individual differences within Asian culture. It is less accurate to conclude that all Asian people mainly endorse a pragmatic attitude toward love. More importantly, Chinese college students have been described as exhibiting high heterogeneity in romantic relationships. For example, previous studies have suggested that Chinese students in university have different goals when building intimate relationships; some of whom may forge relationships out of consideration of marriage in the future, whereas others may only want to fill a spiritual void [15]. Such differences may involve a difference in love attitudes. Similarly, although school education in China encourages a serious attitude toward love [16], it is possible that some students endorse a more playful attitude in romantic relationships. Overall, in addition to exploring differences in love attitudes between Asian and Western culture, it is also valuable to explore individual differences within Asian culture, thereby providing a comprehensive description of love attitudes in this culture. To the best of our knowledge, no previous study has explored typical groups of students who exhibit certain patterns of love attitudes within a single Asian culture.

### The Relationship between Love Attitudes and Other Variables

Previous studies have reported that love attitudes are associated with a wide range of variables, including personality traits [17,18]; life satisfaction [19]; sexual relationships [20]; and psychological stress or disorders [21]. The present study focused mainly on personalities and sexual attitudes because their relationship with love attitudes has been relatively well studied in Western countries but not in China.

Previous studies have well explored the relationship between personality and love attitude using various personality scales [14,22–24]. As mentioned above, we wanted to determine whether some Chinese students do not adhere to the values of serious intimacy encouraged by school education and endorse a more playful attitude; therefore, conscientiousness in the NEO-PI [25], which is related to serious attitude, was considered. Wan et al. [14] surveyed Chinese college students in Hong Kong and found that conscientiousness was negatively associated with ludus and mania, as expected based on the discussion above. However, Cao and Zhang [23] only found a weakly negative association between conscientiousness and mania in Chinese high-school students, and other correlations were non-significant. Such controversial results obtained for a Chinese sample may be due to several factors; one factor worth noting here is that these two studies were only concerned with the general five dimensions in the NEO-PI. Studies using Western samples have demonstrated that relationship patterns with love styles described by subscales of the same dimension vary greatly and that certain subscales could have a significant relationship with love attitudes, whereas others may not. For example, dutifulness and deliberation, which are subscales belonging to dimension of conscientiousness, had a negative association with ludus and mania, and other subscales did not have such associations [26]. Such results have encouraged the exploration of the relationship between personality and love attitudes among Chinese college students described by subdimensions in the NEO-PI.

Sex is an important component in intimate relationships, and the researchers who developed the LAS [8] also developed the Multidimensionality of Sexual Attitudes (MSA) instrument [27] to measure attitudes toward sexual relationships and relevant behaviors. A recent revised version of this scale contained five dimensions: permissiveness (casual sexual behavior), instrumentality (biological or utilitarian understanding of sex), responsibility (birth control and sex education), pleasure (pleasant experience) and communion (idealistic relationship). Permissiveness and instrumentality compose a second-order factor named “sex centered on self”, while responsibility, pleasure and communion compose another second-order factor named “sex centered on the relationship” [28]. Several studies using samples from the Western population have drawn similar results, namely that dimensions belonging to “Sex centered on self” correlate positively with ludus and negatively with agape and that “Sex centered on the relationship” is positively associated with mania and agape [21,27,29–31]. To the best of our knowledge, no study has reported on the relationship between sexual attitudes and love attitudes among Chinese students.

To summarize, although many theories on love attitudes have been put forward in previous studies by using Hendrick and Hendrick’s Love Attitude Scale (LAS) [8], there are two issues that have remained underexplored. First, a further investigation of individual differences in love attitudes, especially among typical groups of people within certain cultural groups, is lacking in previous studies. Second, in terms of Chinese population samples, previous studies on the relationship between the big five personality and love attitudes have produced controversial results and demonstrate a lack of exploration of the subdimensions of personality; moreover, the connection between love attitudes and sexual attitudes has not been confirmed within a Chinese population sample.

To explore individual differences and typical patterns regarding love attitudes among Chinese college students, our essential objective in conducting the present investigation was to study whether students can be categorized into some typical groups according to their love attitudes. Cluster analysis has been identified as an effective way to identify groups of similar individuals when examining multiple constructs of interest [32]. Not only does cluster analysis divide cases into different groups but it also describes the characteristics of certain groups by using patterns of dimensions rather than by simply comparing separate dimensions. Furthermore, sexual attitudes and some interpersonal personality traits were used to test differences among explored clusters to validate the results of cluster analysis and explore the relationship of love attitudes with sexual attitudes and personalities among the Chinese population.

## Method

### Participants

Three hundred and eighty-nine college students were recruited from 10 Universities in Beijing. The sample included 191 (49.1%) females and 198 (50.9%) males, 70 (18.0%) freshmen, 100 (25.7%) sophomore, 113 (29.0%) junior, and 106 (27.2%) senior students.

### Instruments

Data were gathered by administering three instruments, namely, the Love Attitudes Scale (LAS), the Sexual Attitudes Scale and three subdimensions of the NEO-PI.

The Love Attitudes Scale: Short Form was developed by Hendrick, Hendrick and Dicke [33] as a short form of the Love Attitudes Scale [8] to examine the six types of love exhibited by individuals based on Lee’s color of love theory [4]. Its Chinese version was revised by Yang, Bai and Xu [34]. The LAS: Short Form consists of 24 items with a five-point Likert Scale (one = Strongly Agree; five = Strongly Disagree). Four items on the scale represent each of the six major love styles: eros (passionate love), ludus (game-playing love), storge (companionate love), pragma (practical love), mania (possessive, dependent love) and agape (all-giving, selfless love). High scores obtained from each subscale indicate the love attitude of an individual.

The Sexual Attitude Scale (SAS): Multidimensionality of Sexual Attitudes (MSA) [27], revised by Le Gall et al. [28], was used to assess the sexual attitudes of participants, and it was translated into Chinese by He and her colleagues [35]. In this 21-item self-report scale, researchers use a Likert-type response format ranging from one (Strongly Disagree) to five (Strongly Agree). Higher scores reflect a stronger endorsement of corresponding attitudes. For this scale, a two-factor structure has been established, with the subscales labeled as follows: (a) sex centered on self (eight items), which includes two parts, permissiveness (e.g., “one night stand is acceptable”) and instrumentality (e.g., “sex is mainly physiological”); and (b) sex centered on the relationship (eleven items), which includes three parts, responsibility (e.g. “sex education for adolescents is important”), pleasure (e.g., “with the development of a romantic relationship, sex is getting more harmonious”) and communion (e.g., “sex is the most intimate communication between two people.”).

There are three subdimensions of the NEO-PI. The NEO PI-R [25] was translated and revised by Zhang and his team [36]. The NEO PI-R assesses the five domain factors and their 30 facets and comprises 240 items, with eight items to measure each personality facet. We chose three subdimensions: deliberation (C6), dutifulness (C3) and self-discipline (C5) from the conscientiousness domain factor.

### Procedure

The whole procedure was approved by the ethical committee of Department of Psychology, Beijing Normal University. The data collection procedure was conducted in unoccupied classrooms, where students study by themselves, in 10 universities in Beijing, China. The studies took place on a voluntary basis after a brief description of the purpose of the study. The classrooms were able to provide a quiet environment and privacy protection to a certain extent. Details about the importance of providing sincere answers and information about email feedback were explained in an introduction. Therefore, major findings of this study were sent to participants if they left an e-mail address, but no individual report was created because of privacy issue. The consent form including the research purposes, principle of privacy protection and the contact information of the research group members was attached to the first page of the survey questionnaire, but no signed consent form was collected. This was because we believed that this would make participants feel there was a stronger privacy safeguard, and there was also no need to collect these forms except for privacy considerations. Other ethical risks are always low for questionnaire surveys. The whole procedure was also approved by the ethical committee of authors' institution. Data collection was permitted by other universities but no ethical approval was requested because current study was not collaborated with those universities.

### Statistical analysis

As missing data would result in a misleading result for the research, before further statistical analysis, we adopted EM algorithm [37] to replace the vacant statistics in order to deal with missing data. Then, for the present research we conducted the following analysis.

First, to examine psychometric characteristics and correlations of all variables, the mean of each variable and their correlations were calculated. Second, to explore the different patterns of love attitudes and ANOVA, we applied a two-step cluster analysis to the collected data suggested by Gordon [38], using the six subscales (i.e., storge, agape, mania, pragma, ludus and eros) measured by the LAS as grouping variables. To determine the number of clusters, we used SPSS software to conduct hierarchical cluster analysis with Ward’s clustering method [39]. To validate and interpret the profiles of the original cluster solution, we conducted a K-means cluster analytic procedure using the same data. Third, to assess the differences of cluster groups, we compared their demographic variables by *χ*^2^ test and their personalities which are measured by subscales of NEO-PI and SAS by ANOVA.

We analyzed the results using SPSS 19.0. And the data underlying the findings was contained in supporting information S4 Appendix.

## Results

### Psychometric Characteristics and Correlations of All Variables

The mean of each variable and their correlations are listed below in table 1 in supporting information S1 Appendix; the alpha coefficients of each dimension are shown in bold. The reliability of most dimensions is acceptable, although some dimensions showed lower reliability scores, partially due to the low number of items in those subscales. Confirmative factor analysis performed using a model in which the six dimensions of the LAS correlated with each other without second-order factors showed that relative chi-square (*χ2/df*) is 1.859 and RMSEA is .047, which indicated a good fit [40,41]; however, the CFI did not indicate a good fit (.86) [42]. To maintain comparability with other studies, we did not further explore or adjust the structure of the scale. As for the correlations between love attitudes and other variables, sex centered on self had a high correlation with ludus (r = .51) and its correlations with pragma and mania were also significant (r = .10 and .12 respectively), while sex centered on relationship had significant positive correlations with all dimensions of love attitudes (from .13 to .36) except for ludus. Although dutifulness, deliberation and self-discipline are subdimensions of conscientiousness, their correlation patterns with love attitudes were different: dutifulness is significantly correlated with eros, ludus and agape (r = .24, -.24 and .11 respectively), while deliberation had significant correlation with eros and pragma (r = .13 and .30 respectively), and self-discipline had significant correlation with eros, pragma and agape (r = .19, .16 and -.16 respectively).

### Cluster Analysis

We selected cluster analysis to explore the different patterns of love attitudes. The six subscales (i.e., storge, agape, mania, pragma, ludus and eros) measured by the LAS were used as grouping variables. As suggested by Gordon [38], we followed a two-step procedure in exploring cluster groups. First, we used SPSS software to conduct hierarchical cluster analysis with Ward’s clustering method [39] to determine the number of clusters. This method suggested that a four-cluster solution was most appropriate for the data and identified four large unambiguous groups. The numbers of individuals within the clusters, expressed as percentage, were 25.2% (Cluster A), 22.6% (Cluster B), 30.3% (Cluster C) and 21.9% (Cluster D). In the second step, to validate and interpret the profiles of the original cluster solution, we conducted a K-means cluster analytic procedure using the same data. Based on the hierarchical cluster results, we specified four clusters to be identified. The results showed that the hierarchical cluster findings and the K-means cluster findings were highly similar with respect to the size of each cluster, indicating the validity of the findings. The K-means cluster analysis results, including the number of participants in each cluster group and a brief description of each group’s love attitude pattern, are presented in the following paragraphs (see figure 1 in supporting information S3 Appendix). Cluster A—This cluster group was the largest (28.8%). Participants showed relatively higher scores on ludus (*M* = 3.02) and lower scores on agape (*M* = 3.08) and were referred to as “game players”. Cluster B—This cluster (24.2%) was characterized by participants with relatively higher scores on storge (*M* = 3.78) and pragma (*M* = 3.99), who were referred to as “rational lovers”. Cluster C—Participants in this cluster (26.0%) showed relatively low scores on all subscales, especially on pragma (*M* = 2.79), and were referred to as “absence lovers”. Cluster D—Participants in this cluster (21.1%) demonstrated relatively higher scores on eros (*M* = 3.72) and agape (*M* = 3.54), and relatively lower scores on ludus (*M* = 1.80), and were referred to as “emotional lovers”. A further *χ*^*2*^ test showed that there was no significant difference in the proportion of participants between the four groups (*χ*^*2*^ (3) = 4.882, *p* > .05).

### Comparison of Love Attitude Cluster Patterns

#### Demographic variables

We compared the cluster groups of individuals from the present sample with respect to relevant demographic variables. We found no significant differences in gender or grade between the groups (*χ*^*2*^ (3) = 6.022, *p* > .05; *χ*^*2*^ (9) = 3.485, *p* > .05, respectively).

#### Personality

We measured personality by subscales of NEO-PI, the means and standard deviations of cluster groups and the sample were presented in table 2 in supporting information S2 Appendix. The higher means of deliberation, dutifulness and self-discipline indicated higher degree of deliberation, dutifulness and self-discipline.

Using ANOVA, we evaluated personality differences between potential cluster groups. ANOVA revealed that the clusters differed significantly with respect to deliberation (*F* (3, 386) = 8.25, *p* < .001). Post hoc comparisons revealed that rational lovers (*M* = 3.51, *SD* = 0.50) significantly differed from all cluster groups, demonstrating the highest scores, and game players (*M* = 3.34, *SD* = 0.44) significantly differed from absence lovers (*M* = 3.19, *SD* = 0.47). However, there were no significant differences observed between emotional lovers (*M* = 3.23, *SD* = 0.52) and game players, and between emotional lovers and absence lovers.

We also compared the cluster groups with respect to dutifulness. The results indicated that significant differences did exist (*F* (3, 386) = 6.97, *p* < .001). LSD post hoc comparisons revealed that game players (*M* = 3.60, *SD* = 0.43) and absence lovers (*M* = 3.62, *SD* = 0.41) significantly differed from rational lovers (*M* = 3.76, *SD* = 0.39) and emotional lovers (*M* = 3.83, *SD* = 0.41), but there were no differences between game players and absence lovers, and between rational lovers and emotional lovers.

ANOVA revealed no significant differences among clusters with respect to self-discipline (*F* (3, 386) = 2.04, *p* = .108).

#### Sexual attitude

We measured sexual attitudes by SAS, the means and standard deviations of cluster groups and the sample were presented in table 2 in supporting information S2 Appendix. The higher means of sex centered on self and sex centered on the relationship indicated higher tendency to self-centered sex and relationship-centered sex.

The potential differences in sexual attitudes among cluster groups were also measured by SAS and analyzed by ANOVA. (See table 2 in supporting information S2 Appendix).

ANOVA revealed that the clusters differed significantly with respect to the factor sex centered on self (*F* (3, 386) = 12.15, *p* < .001). LSD post hoc comparisons revealed that game players (*M* = 2.81, *SD* = 0.59) significantly differed from all cluster groups, demonstrating the highest scores, and rational lovers (*M* = 2.60, *SD* = 0.61) significantly differed from emotional lovers (*M* = 2.32, *SD* = 0.59). However, there were no significant differences between absence lovers (*M* = 2.47, *SD* = 0.54) and rational lovers, and between absence lovers and emotional lovers. Similarly, we evaluated cluster groups on the sex centered on the relationship factor and observed significant differences (*F* (3, 386) = 14.87, *p* < .001). LSD post hoc comparisons revealed that game players (*M* = 3.44, *SD* = 0.41) and absence lovers (*M* = 3.33, *SD* = 0.36) significantly differed from all cluster groups, with absence lovers demonstrating the lowest scores. However, there were no significant differences between rational lovers (*M* = 3.66, *SD* = 0.35) and emotional lovers (*M* = 3.62, *SD* = 0.45).

## Discussion

### Heterogeneity in Love Attitudes among Chinese Students

We identified four types of students in terms of love attitude via cluster analysis. Based on the pattern of love attitudes, we assumed that different types of students have different purposes or motivations in romantic relationships. Game players were characterized by their relatively higher levels of ludus, suggesting that those students did not have a very serious attitude toward intimacy or considered their intimacy only a game, compared with participants in other groups. Both rational lovers and emotional lovers showed higher levels of eros implying more engagement in love. In comparison, rational lovers showed more storge and pragma, suggesting a more rational consideration for the realistic issue of a long-term relationship. However, emotional lovers showed a higher propensity toward pragma and agape, reflecting greater emotional needs in romantic relationships. The remaining group of participants, absence lovers, showed relatively low scores on all six dimension of the LAS, and it is possible that these students did not have very much interest in romantic relationships at the time of the study. It is worth noticing that the higher or lower scores reflected the relative differences between groups, which did not necessarily indicate absolute preference. For example, the game players had relatively higher score on ludus in comparison with other groups, but their actual mean score was only 3.02 on the 1 to 5 point scale, which indicated that they did not necessarily agree with the descriptions in items. Also, because the absolute scores are influenced by how items express, further investigation is necessary if researchers want to know how exactly students consider certain idea.

The proportion of students in each cluster did not show a significant difference between clusters, which reflects the high heterogeneity of love attitudes among Chinese college students. In particular, consistent with our hypothesis outlined above, although school education in China encourages serious attitudes toward love [16], a quarter of students still endorse a more playful attitude in romantic relationships. It also should be noted that we found no significant differences among the cluster groups in terms of gender and grade, which seems to conflict with the solid conclusion drawn in previous studies that males endorse more ludus than females [8]. Therefore, we conducted a further calculation using the original six dimensions of love attitudes, and the result showed that males scored higher than females on ludus and agape (available upon request), which is consistent with the general tendency reported in previous studies [8]. These findings suggest that, although the cluster analysis was based on the six dimensions of the LAS, the results of cluster analysis provided different information than the data gathered for the six dimensions. As suggested above, the identification of four groups of students reflects the fact that students have different purposes or motivations in romantic relationships that are influenced by many factors. For example, female students could be classified as game players if they did not think a relationship started in university could last after graduation, thereby adopting a playful attitude, and this would not conflict with the fact that their willingness to engage in ludus would be lower than their male counterparts.

### Differences in Personality and Sexual Attitudes by Types of Students

Most differences between types of students with respect to personality were consistent with expectations. The negative association between ludus and the dimensions of conscientiousness reported in previous studies [14,26] was reproduced in our studies. Accordingly, game players, characterized by high ludus, also showed the lowest dutifulness among the four groups. We expected rational lovers to consider more pragmatic factors in romantic relationships, which was consistently reflected in the result that rational lovers showed higher deliberation than emotional lovers. This finding indicates a general tendency to make more comprehensive judgments before taking action. However, it is worth noting that some Western studies did not observe the same relationship between deliberation and pragma [26] that we did. Whether there is a potential cultural difference in this respect needs to be investigated further.

In contrast to our expectations, we did not find significant differences between different types of students with respect to self-discipline, although self-discipline showed a low correlation coefficient with eros, pragma and mania. This result implies the possibility that, although serious commitment in romantic relationships is still encouraged by the education system as a “moral” or “righteous” style of love [16], whether romantic relationships are considered preparation for long-term commitments or considered a game or short-term emotional experience is not a matter of morality or discipline for the new generation of Chinese people. Therefore, self-discipline, which is associated with morality, does not have much of an influence on love attitudes. In addition, self-discipline is more of a personal trait rather than an interpersonal issue. Heaven et al. [26] only selected interpersonal dimensions from the NEO-PI, excluding dimensions such as self-discipline from measurement, and our results also demonstrated that interpersonal dimensions may have a stronger influence on love attitudes than personal dimensions, although more evidence is required to ascertain this conclusion. It is also worth noting that the effect sizes were actually small, which indicated the real differences between groups were not quite large although the significant differences were reached. This is reasonable because the personalities are concerned with overall behaviors and romantic relationship is only a part of life.

With regard to personality traits measured by subdimensions in the NEO-PI, our results demonstrated that different subscales in the same dimensions may indicate different relationship patterns with love attitudes, similar to the results reported in previous studies using Western participants [26]. Such results imply that previous studies with Chinese participants that have reported only a weak relationship between love attitudes and the dimensions of the NEO-PI [23] may in fact be because the general five dimensions diluted or even neutralized the relationship between the subdimensions and love attitudes. Thus, further study is necessary to understand the relationship between personality and love attitudes.

Regarding sexual attitudes, in accordance with the results reported by previous studies using Western participants [27], we found that the sex centered on self factor was closely associated with ludus and that a high score for this factor distinguished game players from other groups. Absence lovers could be clearly distinguished by their lower scores on the sex centered on relationship factor compared with other groups. Because the sex centered on relationship factor connotes that sexual behaviors can bring pleasant experience (i.e., pleasure) and even create an ideal relationship between two people (i.e., communion), this result implies that absence lovers may not link sex with this positive attitude. This finding partially supports our assumption that absence lovers may not have had interest in romantic relationships at the time of this study. Again, the effect sizes for sexual attitudes were also small, which showed that love and sex are two different aspects of close relationship in despite of their associations. Nevertheless, the limited differences in terms of sexual attitudes still validated the results of clusters analysis on love attitudes.

### Limitations and Future Directions

The present research shows some limitations that must be improved upon. Although we made a bold speculation about differences in sexual attitude and personality among the four patterns of love, behavioral characteristics were only measured using self-report scales without obtaining direct proof, such as by recording behavioral modes and conducting further interviews, and the reliability of some inventories and effect size of some variables are relative low. In addition, as we hold the belief that students in college were at the largely same age and age just accounted very small portions for our research question, different patterns of love attitudes among Chinese college students, we did not collect data relevant to age, with which we may be able to draw a more accurate conclusion. Similarly, information about the status of relationship of participants’ parents, their relationship experience and other factors that may interact with our research question, should be collected to get a more comprehensive finding. Furthermore, in the K-means Cluster analysis and hierarchical cluster analysis, our sample was such that hierarchical analysis could not accurately verify the results of K-means analysis and limited the general applicability of the results. Another flaw in this research lies in common method variance [43]. Most researchers agree that common method variance (i.e., variance that is attributable to the measurement method rather than to the constructs the measures represent) is a potential problem in psychological research. Because we used merely questionnaires, further verification must be performed.

Several studies could be conducted in the future. First, the present research found that a quarter of students still endorse a more playful attitude in romantic relationships, this phenomenon is noteworthy for educators in China, as more efforts are needed if educators want to cultivate serious attitudes among students toward their romantic relationships. The current study only pointed out the proportion of game players, further exploration is needed to understand what factors lead to their attitudes and how they consider current education, so that the education strategies could be more effective in the future. Second, cluster analysis with other student samples should be conducted to determine whether the four types of behaviors we identified are stable. In addition, because university students in China are a special group, similar studies with other samples, either university students in other cultures or Chinese people of a different age, would also be valuable. Third, if the four behavior types are confirmed to be stable and typical behavior patterns of university students in China, a number of issues should be further explored, including the reasons that cause the differences in love attitudes between patterns of behavior, the prediction of the propensity for future marriage for different patterns and the differences between current romantic relationships and dating between patterns. In particular, due to the limitation of the length of the questionnaires, this study only chose three subdimensions from the NEO-PI. To gain a better understanding of the difference between patterns in terms of personality, we should also conduct comprehensive measurement based on NEO-PI.

## Supporting Information

S1 Appendix Summary of Intercorrelation, Means and Standard Deviations for Scores on eros, ludus, storge, pragma, mania, agape, sex centered on self, sex centered on the relationship, dutifulness, deliberation and self-discipline. (DOCX)

S2 Appendix Cluster Groups Means and Standard Deviations on the NEO-PI and SAS. (DOCX)

S3 Appendix Different love attitude patterns for K-means cluster groups. (PDF)

S4 Appendix Data underlying the findings. (SAV)
