# Supplementary material for: Exploring Different Patterns of Love Attitudes among Chinese College Students
Source: PLoS One. 2016 Nov 16;11(11):e0166410. doi: 10.1371/journal.pone.0166410 (PMC5113012; doi:10.1371/journal.pone.0166410)
Supplement: S1 Appendix — (DOCX) [file pone.0166410.s001.docx]

Table 1.

*Summary of Intercorrelation, Means and Standard Deviations for Scores on eros, ludus, storge, pragma, mania, agape, sex centered on self, sex centered on the relationship, dutifulness, deliberation and self-discipline*

|  |  | *M* | *SD* | 1 | 2 | 3 | 4 | 5 | 6 | 7 | 8 | 9 | 10 | 11 |
| --- | --- | --- | --- | --- | --- | --- | --- | --- | --- | --- | --- | --- | --- | --- |
| 1 | eros | 3.43 | .60 | **.64** |  |  |  |  |  |  |  |  |  |  |
| 2 | ludus | 2.31 | .76 | -.10 | .**51** |  |  |  |  |  |  |  |  |  |
| 3 | storge | 3.29 | .64 | .15** | -.01 | .**57** |  |  |  |  |  |  |  |  |
| 4 | pragma | 3.32 | .69 | .11* | .19** | .19** | .**66** |  |  |  |  |  |  |  |
| 5 | mania | 3.49 | .64 | .28** | -.01 | .01 | .02 | .**46** |  |  |  |  |  |  |
| 6 | agape | 3.28 | .68 | .31** | -.01 | .20** | -.03 | .32** | .**67** |  |  |  |  |  |
| 7 | sex centered on self | 2.57 | .61 | .04 | .51** | .01 | .10* | .02 | .12* | .**69** |  |  |  |  |
| 8 | sex centered on the relationship | 3.50 | .41 | .36** | -.04 | .13** | .18** | .29** | .31** | .12* | .**66** |  |  |  |
| 9 | dutifulness | 3.69 | .42 | .24** | -.24** | .10 | .08 | .06 | .11* | -.29** | .21** | .**61** |  |  |
| 10 | deliberation | 3.32 | .49 | .13** | -.06 | .06 | .30** | -.06 | -.04 | -.16** | .17** | .31** | .**63** |  |
| 11 | self-discipline | 3.29 | .49 | .19** | -.09 | .09 | .16** | -.16** | -.05 | -.13** | .10 | .41** | .37** | .**66** |

*Note.* Alpha coefficients are in boldface.

*p＜0.05. **p＜0.01.
