# Supplementary material for: Exploring Different Patterns of Love Attitudes among Chinese College Students
Source: PLoS One. 2016 Nov 16;11(11):e0166410. doi: 10.1371/journal.pone.0166410 (PMC5113012; doi:10.1371/journal.pone.0166410)
Supplement: S2 Appendix — (DOCX) [file pone.0166410.s002.docx]

Table2.

*Cluster Groups Means and Standard Deviations on the NEO-PI and SAS (N = 389)*

|  |  | NEP-PI | | | SAS | |
| --- | --- | --- | --- | --- | --- | --- |
|  |  | deliberation | dutifulness | self-discipline | sex centered  on self | sex centered  on the relationship |
| Game Players  (n =112) | M | 3.34 | 3.60 | 3.28 | 2.81 | 3.44 |
|  | SD | 0.44 | 0.43 | 0.49 | 0.59 | 0.41 |
| Rational Lovers  (n =94) | M | 3.51 | 3.76 | 3.40 | 2.60 | 3.66 |
|  | SD | 0.50 | 0.39 | 0.51 | 0.61 | 0.35 |
| Absence Lovers  (n =101) | M | 3.19 | 3.62 | 3.24 | 2.47 | 3.33 |
|  | SD | 0.47 | 0.41 | 0.49 | 0.54 | 0.36 |
| Emotional Lovers  (n =82) | M | 3.23 | 3.83 | 3.26 | 2.32 | 3.62 |
|  | SD | 0.52 | 0.41 | 0.48 | 0.59 | 0.45 |
| Total  (n=389) | M | 3.32 | 3.69 | 3.29 | 2.57 | 3.50 |
|  | SD | 0.49 | 0.42 | 0.49 | 0.61 | 0.41 |
|  | Max | 5.00 | 4.88 | 4.88 | 4.20 | 4.64 |
|  | Min | 1.63 | 2.38 | 1.63 | 1.30 | 2.27 |
|  | F | 8.25^**^ | 6.97^**^ | 2.04 | 12.15^**^ | 14.87^**^ |
|  | p | <.001 | <.001 | 0.108 | <.001 | <.001 |
|  | η^2^ | 0.06 | 0.05 | 0.02 | 0.09 | 0.1 |
|  | LSD | C,D<D,A<B | A,C<B,D | non-sig. | D,C<C,B<A | C<A<B,D |

*Note.* **p＜0.01.
