# Supplementary material for: Exploring Different Patterns of Love Attitudes among Chinese College Students
Source: PLoS One. 2016 Nov 16;11(11):e0166410. doi: 10.1371/journal.pone.0166410 (PMC5113012; doi:10.1371/journal.pone.0166410)
Supplement: S3 Appendix — (PDF) [file pone.0166410.s003.pdf]

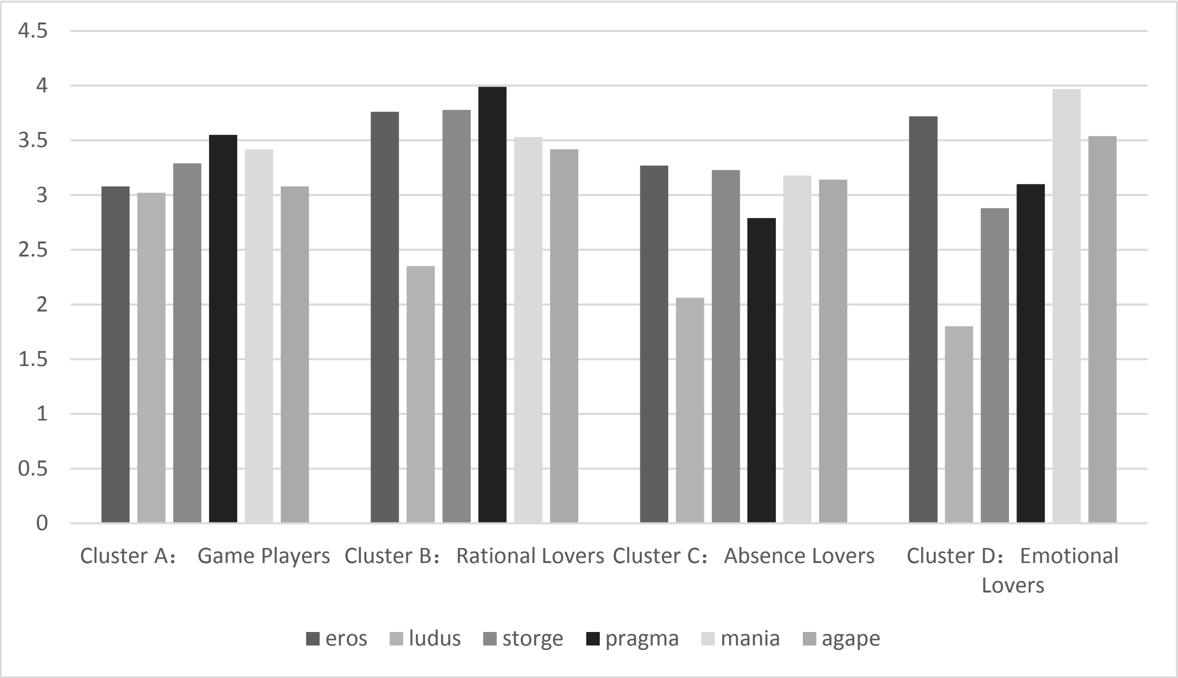

Figure 1. Different love attitude patterns for K-means cluster groups. Bars represent each cluster's scores on six love attitudes and higher scores mean the cluster express more corresponding love attitudes.
